# Stromal Curvature, Power and Corneal‐Stromal Curvature Ratios From a Hybrid AS‐OCT in Eyes With Keratoconus

**DOI:** 10.1111/ceo.70001

**Published:** 2025-09-30

**Authors:** Jascha A. Wendelstein, Annabella Ostermaier, Katrin Freller, Arianna Grendele, Giacomo Savini, Catarina Praefke Coutinho, Robert Herber, Nikolaus Luft, Stefan Kassumeh, Achim Langenbucher, Siegfried Priglinger

**Affiliations:** ^1^ University Eye Hospital Ludwig‐Maximilians‐University Munich Germany; ^2^ Institut Für Refraktive Und Ophthalmo‐Chirurgie (IROC) Zurich Switzerland; ^3^ Institute of Experimental Ophthalmology Saarland University Homburg Germany; ^4^ Department of Ophthalmology and Optometry Steyr General Hospital (LKH Steyr) Steyr Austria; ^5^ Studio Oculistico D'azeglio Bologna Italy; ^6^ IRCCS Bietti Foundation Rome Italy; ^7^ Dipartimento di Farmacia e Biotecnologie University of Bologna Bologna Italy; ^8^ Department of Ophthalmology, Faculty of Medicine and University Hospital Carl Gustav Carus Dresden) Germany

**Keywords:** anterior segment OCT, curvature ratio, keratoconus, MS‐39, refractive surface model, stromal curvature

## Abstract

**Background:**

To characterise stromal curvature and curvature ratios in keratoconus (KCN) using anterior segment OCT, and to evaluate the implications of using single‐, two‐, and three‐surface refractive models for corneal power estimation in ectatic eyes.

**Methods:**

Retrospective observational study. Anterior segment OCT measurements (MS‐39, CSO) were analysed. Anterior, stromal, and posterior curvature radii were computed across five concentric zones (2.0–6.0 mm) using a floating best‐fit sphere, and curvature ratios were subsequently derived: anterior‐to‐stromal (ASR), stromal‐to‐posterior (SPR), and anterior‐to‐posterior (APR). Corneal power was calculated using one‐, two‐, and three‐surface models. KCN severity was classified according to the Belin ABC grading stage and ASR, SPR, and APR were stratified accordingly.

**Results:**

Data from 944 keratoconic eyes were analysed. Peripheral zones (6.0 mm) exhibited reduced variability in curvature measurement compared to central zones (3.0 mm). Differences between simplified (one‐ and two‐surface) and three‐surface power models correlated moderately with increased APR and SPR values. ASR, SPR, and APR all increased progressively with advancing ABC grade.

**Conclusion:**

In advanced keratoconus, three‐surface modelling yields different corneal power estimates versus simplified models in KC; prospective outcome studies are needed to assess clinical impact. Stromal curvature and its derived ratios provide novel structural metrics that change with KCN severity. Curvature ratio increase —especially APR and SPR— reflects posterior steepening and anterior–posterior decoupling, with possible implications for staging and surgical planning.

## 1 Introduction

Accurate characterisation of corneal geometry is essential for the diagnosis and staging of keratoconus (KCN), as well as for individualised refractive and surgical planning. Traditionally, anterior corneal curvature—and more recently, posterior curvature—has served as key parameters for these purposes. However, advances in high‐resolution anterior segment imaging—particularly with optical coherence tomography‐based (OCT) devices—now enable refined, layer‐specific analysis of the cornea.

In our previous work, we introduced a novel three‐layer corneal model based on epithelial mapping and stromal anterior curvature measurements, offering new insights into the optical contributions of the epithelium in four exemplary clinical scenarios (Figure S1) [1, 2]. Additionally, we defined and quantified curvature ratios between individual corneal layers (Figure S1)—namely, the anterior‐to‐stromal ratio (ASR) and the stromal‐to‐posterior ratio (SPR)—and reported normative values for these, along with the anterior‐to‐posterior ratio (APR), across five measurement zones in healthy eyes [3].

Alterations in anterior and posterior curvature are well documented in keratoconic corneas [4]. In healthy eyes, corneal curvature has been shown to depend on axial length (AL) [5]. Nonetheless, contemporary keratoconus grading systems do not incorporate axial length and rely predominantly on fixed keratometric (curvature) cut‐offs [4, 6]. In contrast, curvature ratios such as APR, which are derived from geometric relationships between layers, appear to be independent of AL [3, 5, 7]. As curvature is influenced by AL, AL‐independent ratios may help reduce AL confounding and could be explored as adjunct detection/progression metrics in keratoconus. To date, however, no normative data exist on ASR or SPR in keratoconic eyes, nor is it clear how these values evolve across disease stages.

The current study builds up on previous studies in two ways. First, we assessed the refractive implications of modelling the cornea as a thin lens (single refractive surface), a monolayer structure (two refractive surfaces), or a bilayer structure (three refractive surfaces) in a large cohort of eyes with keratoconus. Second, we establish normative values for ASR, SPR, and APR across five concentric measurement zones (central 2 mm to paracentral 6 mm) in eyes with varying degrees of KCN severity. All data were acquired using the MS‐39 (CSO, Florence, Italy), a combined Placido and OCT‐based tomographer that enables reproducible, layer‐specific curvature assessment. Given the variability in measurement definitions across devices, our results provide device‐specific reference values and highlight how these curvature ratios change with ectatic progression. Model comparisons were performed under identical, literature‐standard refractive index assumptions to isolate geometric effects; hence, outcome‐based validation is deferred to future work.

By characterising these stromal‐based curvature metrics in KCN for the first time, this study sets out to characterise layer‐specific curvature ratios (ASR, SPR, and APR in KCN), quantify how corneal power estimates from simplified 1‐ and 2‐surface models differ from an explicit 3‐surface model, and how these differences scale with the ratios.

## 2 Methods

### 2.1 Data Source and Imaging Protocol

A dataset comprising clinical measurements (*n* = 4940) from consecutive patients with a previous diagnosis of KCN examined at the Department of Ophthalmology, Ludwig‐Maximilians‐University (LMU) Munich, Germany, and the IRCCS Fondazione Bietti, Rome, Italy, was retrospectively screened. All data were anonymised at the source (removing names and birthdates) and compiled in Microsoft Excel (Microsoft Corporation, Redmond, WA, USA). The anonymised dataset was transferred to the Department of Experimental Ophthalmology, Saarland University, for analysis.

All measurements were performed using a high‐resolution anterior segment tomographer (MS‐39, software version 4.1.1), which combines Placido‐based topography with spectral‐domain OCT. Measurement data were obtained using an AS‐OCT (MS‐39, CSO, Italy) and exported with the standard user software. The dataset included corneal curvature, power, and surface height measurements (epithelium, stroma, and endothelium) organised in a cylindrical coordinate system with 256 equidistant semimeridians and 30 radial distances, ranging from 0 mm to 6 mm from the centre in steps of 0.2 mm; no map images or device printouts were used. For spherical fit analysis, all 256 × 30 data points within the 12 mm zone were evaluated. A floating best‐fit sphere was fitted to the epithelium, stroma, and endothelium within central zones of 2.0, 3.0, 4.0, 5.0, and 6.0 mm diameters by minimising the root‐mean‐squared fit error. From these fits, the mean radii of curvature and sphere centres were determined. The extracted radii and apices were then used for paraxial calculations (see Figure S1).

### 2.2 Study Design

This retrospective observational study adhered to the tenets of the Declaration of Helsinki and received ethics approval from the Ärztekammer des Saarlandes/LMU München (approval number 157/21/25–0531). Eyes from patients aged 18 years or older were eligible for inclusion. Only eyes with a confirmed diagnosis of KCN were included, with a minimum requirement of stage 1 in at least one of the A, B, or C parameters of the Belin ABC grading system [4]. Exclusion criteria comprised the presence of anterior (e.g., anterior basement membrane dystrophy) or posterior (e.g., Fuchs endothelial dystrophy) corneal degenerations or dystrophies other than KCN, a history of ocular trauma or corneal scarring, any prior ocular surgery except laser iridotomy or retinopexy, and contact lens wearing during the last week (for soft contact lenses) or month (for gas‐permeable contact lenses) before tomography. Repeated measurements or incomplete or erroneous measurements were excluded (Scans were excluded when the evaluable corneal area was < 85% of the measurement grid or when corneal power was flagged non‐valid for any analysis zone).

### 2.3 Corneal Curvature Analysis and Power Calculation

For curvature analysis, a floating sphere was fit to the height data in terms of minimising the root‐mean‐squared (RMS) fit error to the three corneal surfaces (anterior epithelium, anterior stroma, endothelium). This was performed within five concentric zones of 2.0 mm, 3.0 mm, 4.0 mm, 5.0 mm, and 6.0 mm in diameter. From these fits, radii of curvature and surface apex positions were extracted. All zones were centered on the instrument vertex; no thinnest‐point or posterior‐apex centering was performed. These radii were subsequently used in paraxial vergence equations to calculate corneal power using 1‐, 2‐, and 3‐refractive‐surface (RS) models, as previously described, using refractive indices of 1.000 for air, 1.400 for the epithelium, 1.376 for the stroma and/or entire cornea, and 1.336 for the aqueous humour [1, 3]. All comparisons were performed with fixed, literature‐standard refractive index assumptions across models to isolate geometric differences; index values in KC may deviate from these assumptions. Additionally, a keratometric index of 1.332 referenced to the anterior corneal surface (vertex plane power) was used where applicable. As in our previous studies, all curvature values were expressed as radii in millimetres, where smaller radii represent steeper surfaces, and all ratios represent the relative flatness between two layers [3, 5, 7]. Pachymetry, epithelial thickness, and stromal thickness were defined as the axial apex distance between the respective best‐fit surfaces.

### 2.4 Statistical Analysis

Given the known inter‐eye asymmetry in KCN, both eyes were included where available, and statistical dependencies between fellow eyes were accounted for using linear mixed‐effects models with a random intercept for each patient. This approach allows for valid estimation of fixed effects while adjusting for intra‐subject correlation. Curvature radii, corneal power and layer‐specific curvature ratios (ASR, SPR, APR) were analysed across five concentric zones (2.0 to 6.0 mm). For each zone, we report detailed descriptive statistics including the arithmetic mean, standard deviation (SD), median, interquartile range (IQR) and 95% confidence interval (CI). Furthermore, we stratified the dataset by KCN severity based on the Belin ABC classification system. For the 3.0 mm zone and each severity stage, we report the 2.5th, 5th, 10th, 25th, 75th, and 90th, 95th, and 97.5th percentiles and mean values to reflect the distribution across disease stages.

Exploratory comparisons between groups (e.g., ABC stage strata) were performed using non‐parametric approaches where appropriate. All statistical analyses were conducted using IBM SPSS V29.0.0.0.

## 3 Results

From the original dataset of 4940 measurements (after elimination of repeat measurements, failed measurements, and other exclusion criteria), a final sample of 944 eyes from 519 patients (mean age: 34.13 ± 13.50 years; 31.02% female patients and 67.58% male patients) was extracted for the final analysis. Of these, 94 patients contributed one eye, and 425 patients had bilateral inclusion. The sample included 50.42% left eyes and 49.58% right eyes. Across the ABC grading system, the distribution of eyes per stage was as follows: for parameter A—A0: 529 eyes, A1: 126, A2: 207, A3: 37, and A4: 45; for parameter B—B0: 488 eyes, B1: 72, B2: 178, B3: 55, and B4: 151; and for parameter C—C0: 517 eyes, C1: 245, C2: 157, C3: 24, and C4: 1.

### 3.1 Corneal Curvature Analysis

The anterior corneal surface showed a gradual flattening from the center toward the periphery (mean curvature increasing from 7.228 mm at 2.0 mm to 7.350 mm at 6.0 mm), indicating a prolate profile. The stromal surface also demonstrated a mild prolate trend, with curvature decreasing outward. The posterior surface, while flatter in shape compared to the anterior surface, remained more stable in curvature and also followed a prolate geometry, albeit with lower curvature variation (Table 1). Surface power is presented in Table S1 and corneal thickness in Table S2.

**TABLE 1:** Corneal curvature in all eyes (mm).

Values in mm	Mean	SD	Median	IQR	95% CI lower bound	95% CI upper bound
Anterior						
2.0 mm	7.228	0.600	7.338	0.785	5.830	8.108
3.0 mm	7.248	0.548	7.338	0.697	5.982	8.066
4.0 mm	7.273	0.510	7.353	0.650	6.112	8.042
5.0 mm	7.319	0.461	7.382	0.590	6.283	8.017
6.0 mm	7.350	0.436	7.408	0.549	6.360	8.036
Stromal						
2.0 mm	7.005	0.764	7.126	1.097	5.414	8.229
3.0 mm	7.041	0.661	7.128	0.916	5.663	8.088
4.0 mm	7.074	0.596	7.141	0.829	5.820	8.028
5.0 mm	7.145	0.508	7.200	0.700	6.048	7.976
6.0 mm	7.204	0.465	7.259	0.626	6.190	7.945
Endothelial (posterior)						
2.0 mm	5.802	0.860	5.954	1.333	4.169	7.200
3.0 mm	5.824	0.742	5.963	1.141	4.402	6.960
4.0 mm	5.871	0.655	5.973	0.985	4.592	6.887
5.0 mm	5.979	0.536	6.058	0.758	4.874	6.816
6.0 mm	6.060	0.474	6.120	0.645	5.031	6.814

*Note:* All three surfaces show a prolate trend (flatter periphery), and SD decreases from 2 to 6 mm, indicating more stable curvature estimates in larger zones.

### 3.2 Curvature Ratios

ASR decreased from 1.036 to 1.021 with increasing zone size, suggesting that the prolate geometry is preserved between the anterior and stromal layers, as both flatten outward. SPR also showed a decreasing trend (1.261 to 1.215), although the posterior surface flattens less than the stroma, particularly in more central zones.

**FIGURE 1:**
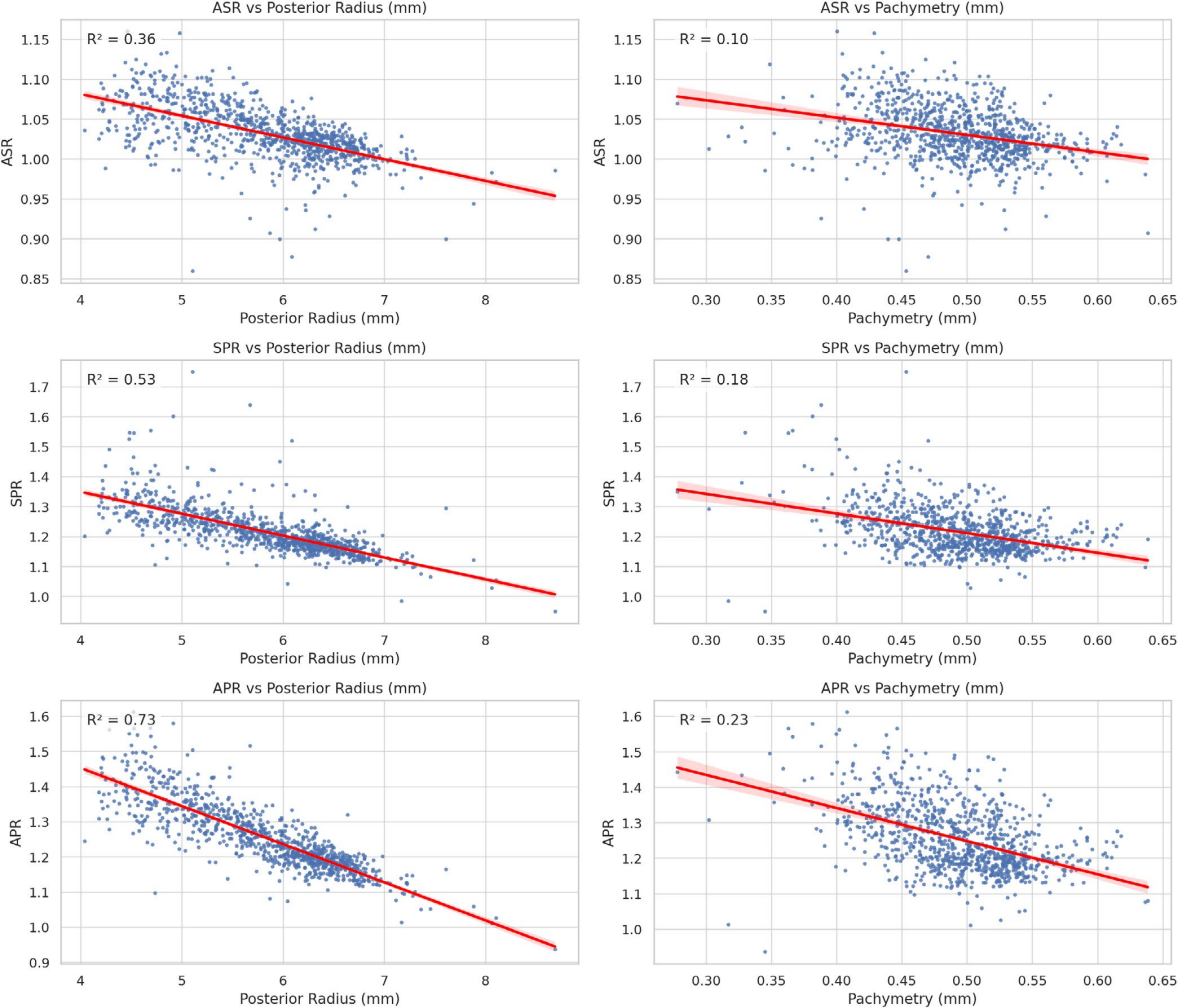
Scatter plots of curvature ratios (ASR, SPR, APR) versus posterior corneal radius and pachymetry, with linear regression lines and *R* ^2^ values. Each panel shows the relationship between a curvature ratio—Anterior‐Stromal Ratio (ASR), Stromal‐Posterior Ratio (SPR), or Anterior–Posterior Ratio (APR)—and either the posterior corneal radius (left column) or central pachymetry (right column). Red lines indicate the best‐fit linear regression, and *R* ^2^ values quantify the proportion of variance in the ratio explained by each parameter. Key finding: ASR, SPR, and APR decline with zone diameter and show lower dispersion at 6 mm, suggesting greater measurement stability in peripheral zones.

APR values followed the same pattern, reinforcing the overall prolate nature of the full cornea (Table 2). SD decreased with measurement zone. Relationships with pachymetry and posterior radius are shown in Figure 1; especially the three plots on the left side show a large amount of heteroscedasticity.

**TABLE 2:** Curvature ratios ASR, Anterior‐Stromal Ratio; SPR, Stromal‐Posterior Ratio; APR, Anterior–Posterior Ratio in all eyes.

	Mean	SD	Median	IQR	95% CI lower bound	95% CI upper bound
Anterior‐Stromal Ratio						
2.0 mm	1.036	0.045	1.028	0.055	0.960	1.135
3.0 mm	1.032	0.034	1.028	0.039	0.972	1.102
4.0 mm	1.030	0.028	1.027	0.033	0.979	1.087
5.0 mm	1.025	0.020	1.024	0.023	0.988	1.066
6.0 mm	1.021	0.015	1.019	0.017	0.991	1.052
Stromal‐Posterior Ratio						
2.0 mm	1.261	0.121	1.233	0.156	1.083	1.546
3.0 mm	1.255	0.094	1.234	0.128	1.122	1.471
4.0 mm	1.246	0.077	1.232	0.103	1.136	1.422
5.0 mm	1.228	0.054	1.218	0.072	1.149	1.350
6.0 mm	1.215	0.043	1.208	0.055	1.149	1.311
Anterior–Posterior Ratio						
2.0 mm	1.217	0.097	1.198	0.105	1.081	1.456
3.0 mm	1.216	0.075	1.200	0.084	1.117	1.384
4.0 mm	1.210	0.061	1.199	0.071	1.127	1.347
5.0 mm	1.198	0.044	1.191	0.052	1.133	1.304
6.0 mm	1.191	0.037	1.186	0.042	1.137	1.280

*Note:* ASR, SPR, and APR decline with zone diameter and show lower dispersion at 6 mm, suggesting greater measurement stability in peripheral zones.

### 3.3 Corneal Power Analysis

**FIGURE 2:**
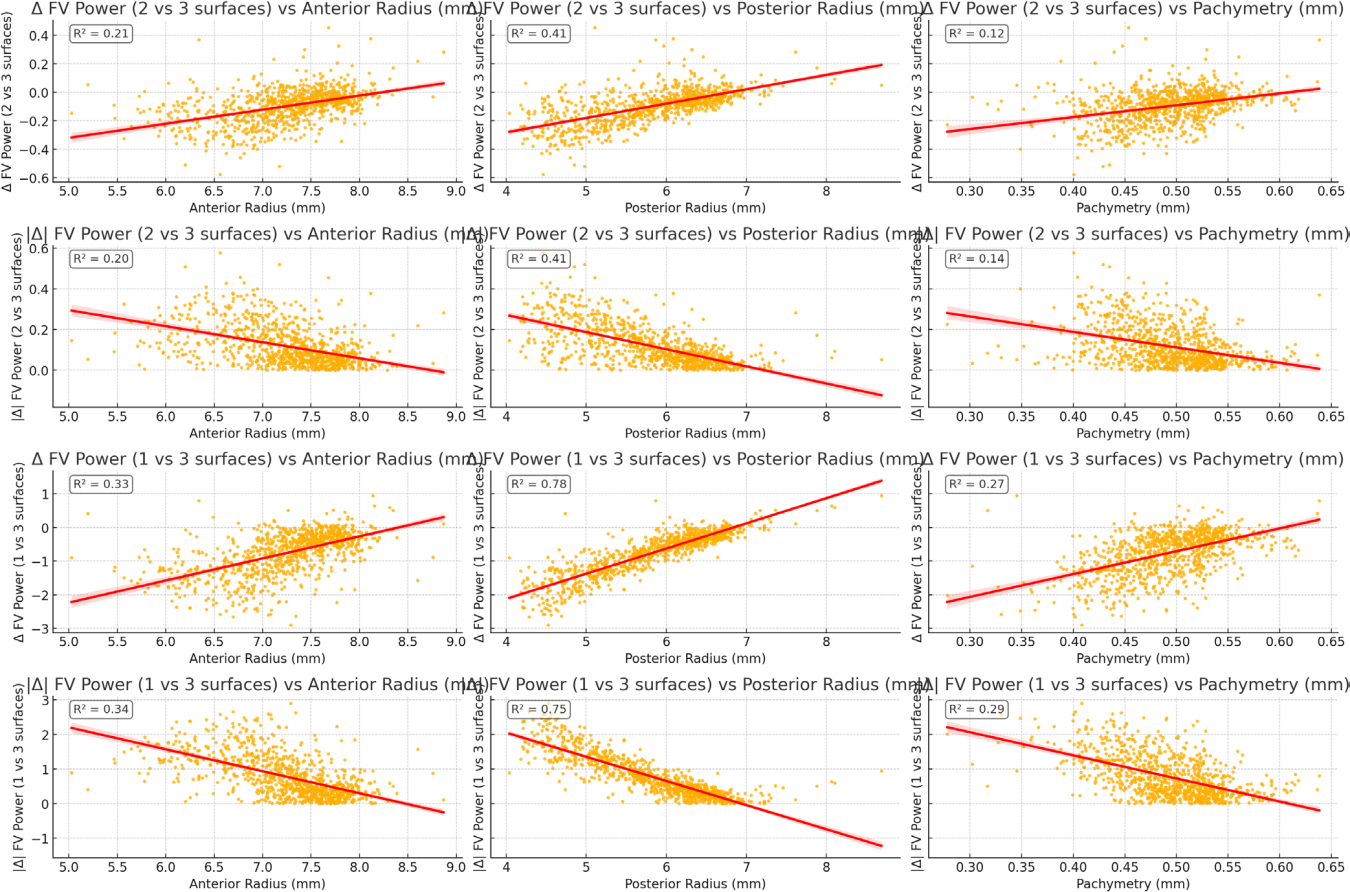
Each subplot shows: The difference (Δ) or absolute difference (|Δ|) in corneal power (referenced to the front vertex FV) between simplified models and the full 3‐surface model. Relationships with anterior radius, posterior radius, and pachymetry are shown. Annotated *R* ^2^ values quantify the strength of each correlation. All plots show a rather high heteroscedasticity. Key finding: Δ (1–3) exceeds Δ (2–3) and shows modest, heteroscedastic relationships with anterior/posterior radius and pachymetry—emphasising that simplified‐versus‐3‐surface differences vary widely across ectatic geometries.

Power calculated from the 1‐surface model overestimates central power due to the inability to capture the true posterior contribution—especially in eyes with steeper anterior and/or posterior corneal curvature (Figure 2).

**FIGURE 3:**
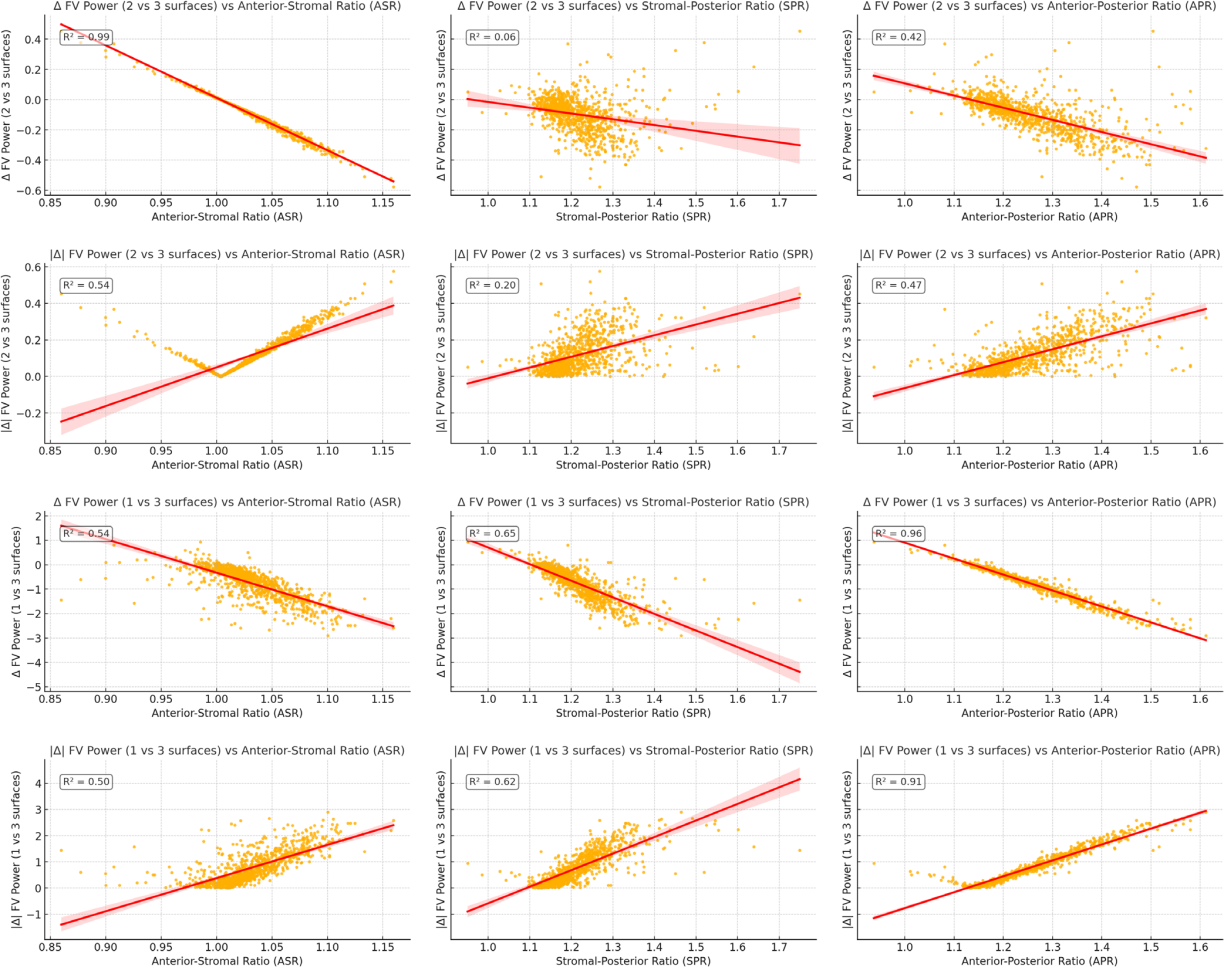
Scatter plots of differences between corneal power models (2 vs. 3 surfaces and 1 vs. 3 surfaces) plotted against the curvature ratios; ASR (Anterior‐Stromal Ratio), SPR (Stromal‐Posterior Ratio), APR (Anterior–Posterior Ratio). Each subplot includes a linear regression line (red) and an annotated *R* ^2^ value showing the strength of the relationship. Key finding: As APR gets larger, the gap between the 1‐surface and 3‐surface power grows. There's a simple tipping point around APR ≈ 1.15: Above ~1.15 the 1‐surface model tends to overestimate corneal power, below ~1.15 it tends to underestimate corneal power. As SPR gets larger, the gap between the 2‐surface and 3‐surface power grows. There's a simple tipping point around SPR ≈ 1.0: Above ~1.00 the 2‐surface model tends to overestimate corneal power, below ~1.00 it tends to underestimate corneal power.

The 2‐ and 3‐surface models capture more of the stromal and posterior steepening, especially as keratoconus advances. As seen in the data, all models showed declining power toward the periphery, confirming an overall prolate pattern (Table 3, Figure 3).

**TABLE 3:** Corneal power in all eyes.

Values in dioptres	Refractive surfaces	Mean	SD	Median	IQR	97.5% Quantile	2.5% Quantile
2.0 mm	1	46.277	4.213	45.244	4.940	40.946	56.950
	2	45.574	3.924	44.611	4.131	40.664	55.768
	3	45.462	3.868	44.548	4.037	40.667	55.551
3.0 mm	1	46.085	3.762	45.244	4.365	41.161	55.498
	2	45.425	3.495	44.634	3.883	40.794	54.405
	3	45.327	3.454	44.526	3.801	40.705	54.220
4.0 mm	1	45.886	3.445	45.149	4.049	41.281	54.324
	2	45.279	3.223	44.626	3.692	40.950	53.231
	3	45.190	3.189	44.552	3.652	40.881	53.131
5.0 mm	1	45.553	3.042	44.972	3.633	41.414	52.841
	2	45.054	2.901	44.533	3.400	40.962	52.006
	3	44.981	2.881	44.485	3.343	40.922	51.909
6.0 mm	1	45.336	2.854	44.814	3.353	41.312	52.197
	2	44.908	2.759	44.483	3.142	41.019	51.547
	3	44.851	2.747	44.424	3.125	40.965	51.461

*Note:* Presented are 1 refractive surface, 2 refractive surfaces and 3 refractive surfaces models. All 3 models are referenced to the front vertex plane to be inline for example, to the standards of biometric measurements for IOL power calculation (D). Key finding: 1‐surface power > 2‐surface > 3‐surface centrally, with all models decreasing peripherally—quantifying the systematic overestimation from simplified models in ectatic corneas.

Power differences between simplified and full models increased as curvature ratios (particularly APR and SPR) rose (Figures 2 and 3).

### 3.4 Curvature Ratios Across ABC Grading

In early keratoconus (A1–A2, B1–B2), both ASR and APR increase subtly—reflecting a relative steepening of the anterior and posterior surfaces, preserving a prolate contour.

In advanced stages (A3–A4, B3–B4), the posterior surface often steepens disproportionately while the anterior becomes relatively flatter or irregular.

**FIGURE 4:**
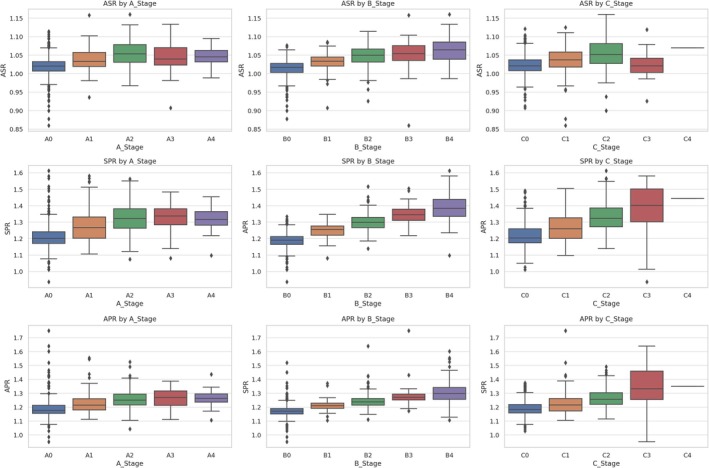
Boxplots of curvature ratios (ASR, SPR, APR) across Belin ABC grading stages (A–C). Each subplot displays the distribution of one curvature ratio—Anterior‐Stromal Ratio (ASR), Stromal‐Posterior Ratio (SPR), and Anterior–Posterior Ratio (APR)—stratified by A‐stage (anterior radius), B‐stage (posterior radius), and C‐stage (pachymetry). In each boxplot, the box represents the interquartile range (IQR), spanning the 25th to 75th percentile of the data. The horizontal line inside the box marks the median (50th percentile). The whiskers extend to the most extreme values within 1.5× IQR from the box edges. Data points beyond the whiskers are plotted as individual dots and represent potential outliers. Key finding: ASR/SPR/APR rise progressively with ABC severity and the spread widens in advanced stages (e.g., B3–B4/C3), capturing the increasing posterior‐dominant imbalance and heterogeneity in advanced KCN.

This is captured in the elevation of SPR and APR in later stages (Table 4, Figures 1 and 4). The increased spread and outliers also reflect irregular surface changes—another hallmark of an oblate or distorted profile.

**TABLE 4:** ASR, SPR, APR in various stages of the Belin Ambrosio ABC grading (which is defined for a 3 mm measurement zone).

	2.5% Quantile	5% Quantile	10% Quantile	25% Quantile	50% Quantile	75% Quantile	90% Quantile	95% Quantile	97.5% Quantile	Mean
Anterior‐Stromal Ratio										
A0	0.960	0.981	0.990	1.007	1.020	1.032	1.051	1.066	1.079	1.020
**A1**	**0.987**	**0.993**	**1.000**	**1.019**	**1.033**	**1.057**	**1.074**	**1.092**	**1.101**	**1.037**
**A2**	**0.987**	**0.998**	**1.010**	**1.030**	**1.053**	**1.078**	**1.099**	**1.108**	**1.115**	**1.052**
A3	0.985	0.992	1.003	1.023	1.048	1.073	1.088	1.093	1.106	1.048
A4	0.994	1.005	1.022	1.032	1.045	1.063	1.076	1.081	1.082	1.046
B0	0.963	0.979	0.989	1.003	1.017	1.028	1.038	1.046	1.053	1.014
**B1**	**0.980**	**0.993**	**1.008**	**1.020**	**1.033**	**1.045**	**1.062**	**1.074**	**1.083**	**1.032**
**B2**	**0.989**	**0.997**	**1.010**	**1.031**	**1.050**	**1.066**	**1.081**	**1.094**	**1.102**	**1.047**
B3	0.995	1.011	1.017	1.036	1.054	1.076	1.086	1.094	1.101	1.051
B4	1.000	1.011	1.020	1.038	1.064	1.085	1.104	1.111	1.122	1.063
C0	0.971	0.983	0.991	1.008	1.021	1.037	1.060	1.073	1.083	1.023
**C1**	**0.977**	**0.991**	**1.001**	**1.018**	**1.037**	**1.058**	**1.078**	**1.085**	**1.101**	**1.037**
**C2**	**0.981**	**0.997**	**1.012**	**1.027**	**1.052**	**1.081**	**1.101**	**1.108**	**1.118**	**1.053**
**C3**	**0.960**	**0.986**	**0.987**	**1.003**	**1.021**	**1.042**	**1.072**	**1.078**	**1.096**	**1.023**
C4	1.070	1.070	1.070	1.070	1.070	1.070	1.070	1.070	1.070	1.070
Stromal‐Posterior Ratio										
A0	1.109	1.122	1.134	1.155	1.177	1.212	1.245	1.282	1.368	1.190
**A1**	**1.137**	**1.141**	**1.155**	**1.179**	**1.214**	**1.259**	**1.310**	**1.360**	**1.434**	**1.229**
**A2**	**1.148**	**1.158**	**1.173**	**1.211**	**1.249**	**1.293**	**1.332**	**1.354**	**1.388**	**1.254**
A3	1.123	1.159	1.186	1.242	1.281	1.323	1.355	1.369	1.383	1.274
A4	1.170	1.176	1.203	1.235	1.262	1.295	1.326	1.332	1.343	1.262
B0	1.108	1.119	1.130	1.151	1.170	1.190	1.215	1.234	1.255	1.172
**B1**	**1.149**	**1.162**	**1.173**	**1.190**	**1.210**	**1.228**	**1.245**	**1.264**	**1.287**	**1.211**
**B2**	**1.168**	**1.173**	**1.190**	**1.214**	**1.237**	**1.262**	**1.282**	**1.314**	**1.373**	**1.241**
**B3**	**1.196**	**1.208**	**1.224**	**1.251**	**1.270**	**1.293**	**1.317**	**1.329**	**1.395**	**1.280**
B4	1.186	1.212	1.235	1.255	1.298	1.340	1.388	1.437	1.530	1.305
C0	1.119	1.126	1.139	1.158	1.182	1.218	1.259	1.282	1.319	1.192
**C1**	**1.111**	**1.122**	**1.139**	**1.173**	**1.215**	**1.261**	**1.301**	**1.330**	**1.373**	**1.223**
**C2**	**1.144**	**1.160**	**1.172**	**1.219**	**1.255**	**1.304**	**1.352**	**1.393**	**1.427**	**1.263**
C3	0.970	1.013	1.171	1.254	1.333	1.459	1.551	1.594	1.617	1.345
C4	1.349	1.349	1.349	1.349	1.349	1.349	1.349	1.349	1.349	1.349
Anterior‐Posterior Ratio										
A0	1.117	1.127	1.142	1.170	1.200	1.241	1.300	1.332	1.379	1.213
**A1**	**1.142**	**1.148**	**1.171**	**1.201**	**1.265**	**1.331**	**1.396**	**1.442**	**1.538**	**1.275**
**A2**	**1.143**	**1.176**	**1.203**	**1.260**	**1.319**	**1.379**	**1.445**	**1.471**	**1.497**	**1.320**
A3	1.174	1.185	1.265	1.296	1.345	1.385	1.401	1.442	1.480	1.335
A4	1.218	1.225	1.241	1.281	1.317	1.364	1.397	1.421	1.437	1.320
B0	1.101	1.123	1.135	1.164	1.190	1.211	1.240	1.263	1.282	1.188
**B1**	**1.157**	**1.180**	**1.193**	**1.220**	**1.256**	**1.276**	**1.310**	**1.330**	**1.336**	**1.250**
**B2**	**1.207**	**1.230**	**1.242**	**1.266**	**1.298**	**1.329**	**1.356**	**1.385**	**1.426**	**1.300**
B3	1.232	1.251	1.278	1.311	1.344	1.378	1.401	1.437	1.473	1.344
B4	1.243	1.273	1.297	1.335	1.383	1.438	1.491	1.545	1.566	1.387
C0	1.117	1.132	1.146	1.174	1.203	1.259	1.321	1.357	1.384	1.220
**C1**	**1.122**	**1.135**	**1.163**	**1.201**	**1.259**	**1.326**	**1.387**	**1.424**	**1.455**	**1.268**
**C2**	**1.165**	**1.193**	**1.220**	**1.271**	**1.322**	**1.386**	**1.440**	**1.485**	**1.521**	**1.330**
C3	0.981	1.038	1.194	1.302	1.401	1.501	1.561	1.577	1.580	1.375
C4	1.443	1.443	1.443	1.443	1.443	1.443	1.443	1.443	1.443	1.443

*Note:* ASR, SPR, APR in various stages of the Belin Ambrosio ABC grading (which is defined for a 3 mm measurement zone). The case numbers for each grading stage were: A0 = 529; A1 = 126; A2 = 207; A3 = 37; A4 = 45; B0 = 488; B1 = 72; B2 = 178; B3 = 55; B4 = 151; C0 = 517; C1 = 245; C2 = 157; C3 = 24; C4 = 1. Bold values differ statistically significantly from the respective preceding stage.

## 4 Discussion

In this MS‐39 cohort of KCN eyes, layer‐specific curvature ratios (ASR/SPR/APR) increased with disease, reflecting anterior–posterior imbalance. Relative to an explicit three‐surface model, power estimates from simplified one‐ and two‐surface models diverged more in KCN than expected in normal eyes, and these divergences increased with APR and SPR (Figures 2 and 3). We also observed zone‐dependent pivot behaviour: corneal power differences between one‐ and three‐surface models changed sign near APR ≈ 1.15, and corneal power differences between two‐ and three‐surface models changed sign near SPR ≈ 1.0. These are descriptive, device/zone‐specific observations, not clinical thresholds.

This study provides the first large‐scale analysis of stromal curvature and its associated curvature ratios—ASR, SPR, and APR—in keratoconic eyes using an AS‐OCT device. Here, ASR/SPR/APR function as layer‐imbalance descriptors rather than levers for power calculation. Because they are dimensionless and AL‐independent, they may complement current staging by flagging deeper‐layer‐driven change and by providing progression metrics once test–retest limits and stage‐specific reference percentiles are established.

Our findings underscore several optical trends that characterise the progression of keratoconus and refine our understanding of corneal remodelling in this disease.

ASR may be useful for identifying early stromal changes even when anterior topography appears regular. Since smaller radii indicate steeper curvature, a lower ASR suggests that the stroma is relatively steeper than the epithelium, whereas a higher ASR indicates a flatter stroma in relation to the epithelium. Compared to healthy eyes, KCN eyes exhibit both higher mean values and nearly twice the standard deviation for the ASR, SPR, and APR ratios [3]. For example, at the 3 mm zone, the mean ASR, SPR, and APR in healthy eyes are 1.007, 1.162, and 1.170, respectively, while in KCN eyes, these increase to 1.032, 1.255, and 1.216. A similarly elevated APR has been previously reported [8, 9]. As APR compares anterior to posterior curvature, higher values indicate a relatively flatter anterior surface, while lower values reflect a steeper one. Even KCN eyes in stages A0, B0, or C0, but especially beginning at stages A1, B1, or C1, show increased curvature ratios from healthy eyes (Table 4). These increased ratios across all three surfaces primarily reflect posterior corneal steepening—a hallmark of keratoconus—observable in both early and advanced stages [6, 10, 11, 12].

Many KCN staging systems rely solely on corneal curvature rather than considering curvature imbalances [4, 6]. Since assessments of KCN progression typically include only tomographic measurements without incorporating ocular biometry, data on the relationship between corneal curvature and AL in KCN eyes remain limited. However, in normal eyes, it has been shown in a large dataset of 10,000 eyes that corneal curvature is strongly dependent on AL and therefore must be interpreted in the context of AL [5]. In comparison, in an even larger dataset comprising over 110,000 eyes, the APR appeared to be independent of AL [5, 7]. Therefore, staging systems based on corneal curvature or Kmax could be refined by incorporating AL, or alternatively, by shifting to markers that are independent of AL, such as ASR, SPR, or APR. Stromal curvature ratios reflect distinct stages of ectatic progression; hence, the curvature ratios ASR, SPR, and APR all showed consistent and progressive changes across the Belin ABC grading system, indicating their potential utility as quantitative markers of disease severity. In early disease stages, a rising ASR may either reflect true steepening of the anterior stromal surface or compensatory epithelial flattening that masks underlying ectasia [13]. In addition, the distribution of epithelial thickness becomes more inhomogeneous with increasing severity of KCN, as the epithelial layer increasingly smoothes the anterior corneal surface. Therefore, the actual stromal curvature may be of greater clinical benefit in the treatment of KCN, as well as other corneal diseases [12, 14]. With respect to the relationship between each ratio and the anterior corneal radius (A stage), the stability of these ratios in stages A2 and A4 (as shown in Figure 4) suggests that epithelial remodelling is active in the early stages of the disease—when the anterior corneal radius tends to remain stable—but diminishes as ectasia progresses.

The SPR and the APR both showed a more pronounced increase with disease severity. Given that smaller posterior radii correspond to steeper surfaces, higher SPR and APR values reflect a disproportionate steepening of the posterior surface relative to the stromal and anterior surfaces [8, 9], or may reflect eccentric posterior steepening, commonly associated with cone decentration in non‐central keratoconus phenotypes. These changes were most prominent in grade C3 eyes, which exhibited the highest mean values and widest quantile spreads, suggesting significant interindividual variability in advanced disease. In early keratoconus, the corneal profile remains globally prolate, with curvature gradually decreasing from centre to periphery and curvature ratios reflecting a relatively balanced geometry between layers. As disease progresses, curvature ratios rise due to increasing steepness of deeper surfaces (stromal and posterior), reflecting asphericity and shape distortion. In advanced keratoconus, particularly in grades B3–B4 and C3, this leads to a shift toward a more irregular configuration, driven by pronounced posterior steepening while the anterior surface may appear flatter or irregular depending on epithelial remodelling. The variability of curvature ratios in these stages captures the heterogeneity in ectatic presentation.

These findings underscore the importance of posterior curvature analysis in keratoconus staging. Unlike anterior topography alone, curvature ratios based on radii offer a layer‐specific view of the structural remodelling that occurs during disease progression. Importantly, rising SPR or APR in the absence of pachymetric progression may offer early signs of ectatic destabilisation of corneal optics, potentially preceding changes in Kmax or thinnest pachymetry. The marked increase in posterior steepening—particularly relative to stromal curvature—may serve as an early indicator of ectatic geometric remodelling/irregularity. Future research should focus on validating these curvature ratio metrics in larger datasets and exploring their prognostic value for progression and treatment response.

Peripheral zones offer lower variability but require recalibration: An important observation was the decreasing standard deviation of curvature ratios in more peripheral zones (notably at 6.0 mm). This suggests that larger zones may yield more stable measurements, potentially increasing the reliability of curvature‐based diagnostics. Since irregularities in keratoconus are usually decentered, peripheral sampling may better capture ectatic distortion than central 3.0 mm measurements. However, these peripheral ratios consistently differed in absolute values from the 3.0 mm reference zone, underscoring that current AB grading thresholds are not directly transferable. Before 6.0 mm zone values can be clinically integrated, new normative datasets and cutoffs must be defined to avoid misclassification. E for Epithelium—or better stromal curvature—could be included in the grading.

The observed differences of corneal power when comparing one‐surface and two‐ (or three‐) surface cornea models in KCN have been previously reported when comparing one‐surface calculations to two‐surface calculations [15]. These findings affirm that simplified models inadequately account for posterior steepening and stromal changes in ectatic eyes, especially in further progressed stages (and therefore increasing APR and SPR). Accordingly, IOL power calculations in KCN still show higher variance than in normal eyes [16]. The integration of the three‐layer cornea model is a candidate to test for accuracy in IOL calculation or refractive surgery in future work [2, 16, 17, 18], [15, 16].

Another technical consideration concerns the exclusion of a substantial number of cases due to low measurement quality, which underscores the inherent challenge of imaging highly irregular corneas, particularly in advanced keratoconus. Although the MS‐39 employs a dense azimuthal sampling (typically 256 meridians over 360°), the radial resolution remains limited, with a default spacing of 0.2 mm and 265 sampling points. In ectatic eyes, where surface irregularity and decentered cones are common, this radial resolution may be insufficient, leading to increased measurement noise and reduced reliability of curvature fitting. Future analyses may benefit from a denser radial sampling grid to improve the robustness of curvature estimation and reduce data exclusion in advanced keratoconus.

Limitations: Several limitations must be acknowledged. First, the study's retrospective nature and reliance on a single imaging device (MS‐39) limit generalisability. Second, some ABC grading categories—particularly C4—were underrepresented, limiting conclusions for extreme pachymetric thinning. Furthermore, we lack a keratoconus pattern classification, as we did not separate cases with central steepening from cases with peripheral steepening. Additionally, while curvature ratios provide robust structural markers, they do not capture all aspects of ectatic loss of corneal geometry or epithelial compensation, and should be interpreted alongside other diagnostic parameters. We did not perform test–retest measurements in this cohort. Published data indicate that repeatability declines with keratoconus severity—notably for curvature/elevation—while epithelial/pachymetric metrics remain comparatively repeatable but device‐dependent and not interchangeable; thus absolute values are platform‐ and stage‐dependent [19, 20, 21, 22]. Finally, our curvature analysis was based on best‐fit floating spheres. Although this approach simplifies interpretation and allows consistent comparison across layers, alternative surface fits—such as floating conoids or aspheres—may better reflect the true geometry of keratoconic corneas, especially in advanced or asymmetric cases. However, such modelling adds complexity and remains beyond the scope of the present study. We did not separate central‐cone from peripheral/pellucid‐like phenotypes and did not re‐centre zones on thinnest pachymetry or posterior apex. These phenotype‐stratified and re‐centred analyses are important and will be addressed in follow‐up work.

Outlook: Based on these descriptive data, two avenues merit prospective evaluation. First, in atypical/special eyes (e.g., KCN), the clinical value of explicit three‐surface corneal models for IOL power calculation should be tested against standard single–/two‐surface approaches (the same goes for, where feasible, full‐aperture ray tracing) with postoperative refraction as the accuracy endpoint. Second, because ASR/SPR/APR are dimensionless and AL‐independent, they warrant evaluation as adjunct markers for grading and progression. Given the trade‐off between familiarity at 3 mm (aligns with ABC) and lower dispersion at 6 mm (greater stability), future work should define zone‐ and device‐specific reference percentiles, repeatability, and progression cutoffs.

## Disclaimer

The authors did not receive financial support for the research, authorship, and/or publication of this article. No benefits in any form have been received or will be received from a commercial party related directly or indirectly to the subject of this article.

## Conflicts of Interest

Dr. Wendelstein reports research support from Carl Zeiss Meditec A.G. He reports personal fees from Alcon Surgical, Bausch and Lomb, Carl Zeiss Meditec A.G., Heidelberg Engineering, Rayner Surgical, and Johnson & Johnson Vision outside of the submitted work. He was supported by an “ESCRS Peter Barry Fellowship Grant”. Dr. Herber reports speaker's fee from Heidelberg Engineering and Oculus Optikgeraete. Dr. Langenbucher reports personal fees from Bausch & Lomb and Johnson & Johnson Vision outside the submitted work. Dr. Savini has received personal fees from Alcon, Moptim, SIFI, Thea and Zeiss. There are no conflicts of interest for the other authors.

## Supporting information


**Figure S1:** Schematic scheme modelling the cornea as a monolayer structure two refractive surfaces, (a), or a bilayer structure three refractive surfaces, (b). Anterior to posterior curvature ratio (APR) is displayed by surfaces R1/R3, and anterior to stromal curvature ratio (ASR) and stromal to posterior curvature ratio (SPR) are displayed by surfaces R1/R2 and R2/R3, respectively. R1: Epithelial front radius of curvature; R2: Stromal front radius of curvature; R3: Corneal back surface radius of curvature; n1: refractive index of air; n2: refractive index of epithelium; n3: refractive index of stroma/corneal tissue; n4: refractive index of aqueous; T1: epithelial thickness; T2: stromal thickness.

**Table S1:** Front, stromal and back surface power in all eyes (D). The anterior surface power decreased from center to periphery (from 55.76 D at 2.0 mm to 54.62 D at 6.0 mm), consistent with a prolate anterior power profile.

**Table S2:** Central corneal (C), stromal (S), and epithelial (E) thickness in all eyes (mm). Central corneal, central stromal, and central epithelial thicknesses were consistent across zones.

## Data Availability

The data that support the findings of this study are available from the corresponding author upon reasonable request.
