# Supplementary figures and images for: Stromal Curvature, Power and Corneal‐Stromal Curvature Ratios From a Hybrid AS‐OCT in Eyes With Keratoconus
Source: Clin Exp Ophthalmol. 2025 Sep 30;54(1):9–20. doi: 10.1111/ceo.70001 (PMC12886616; doi:10.1111/ceo.70001)

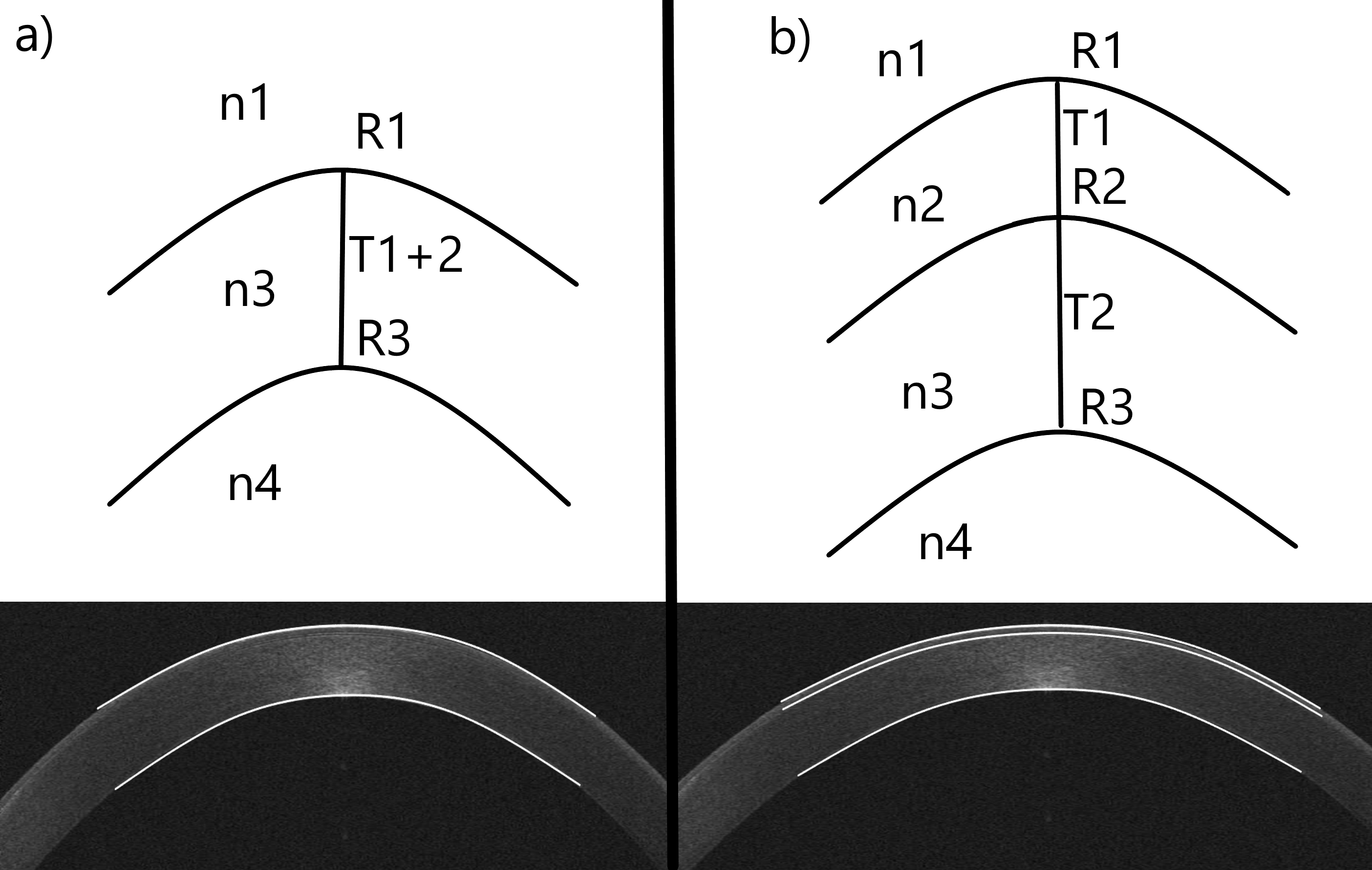

Supplement: Supplementary file 1 — Figure S1: Schematic scheme modelling the cornea as a monolayer structure two refractive surfaces, (a), or a bilayer structure three refractive surfaces, (b). Anterior to posterior curvature ratio (APR) is displayed by surfaces R1/R3, and anterior to stromal curvature ratio (ASR) and stromal to posterior curvature ratio (SPR) are displayed by surfaces R1/R2 and R2/R3, respectively. R1: Epithelial front radius of curvature; R2: Stromal front radius of curvature; R3: Corneal back surface radius of curvature; n1: refractive index of air; n2: refractive index of epithelium; n3: refractive index of stroma/corneal tissue; n4: refractive index of aqueous; T1: epithelial thickness; T2: stromal thickness. [file CEO-54-9-s003.tif]
