# Supplementary material for: Stromal Curvature, Power and Corneal‐Stromal Curvature Ratios From a Hybrid AS‐OCT in Eyes With Keratoconus
Source: Clin Exp Ophthalmol. 2025 Sep 30;54(1):9–20. doi: 10.1111/ceo.70001 (PMC12886616; doi:10.1111/ceo.70001)
Supplement: Supplementary file 2 — Table S1: Front, stromal and back surface power in all eyes (D). The anterior surface power decreased from center to periphery (from 55.76 D at 2.0 mm to 54.62 D at 6.0 mm), consistent with a prolate anterior power profile. [file CEO-54-9-s002.docx]

| **Values in dioptres** | **Mean** | **SD** | **Median** | **IQR** | **95%CI lower bound** | **95%CI upper bound** |
| --- | --- | --- | --- | --- | --- | --- |
| anterior | | | | | | |
| 2.0 mm | 55.755 | 5.076 | 54.511 | 5.951 | 49.333 | 68.614 |
| 3.0 mm | 55.524 | 4.532 | 54.511 | 5.259 | 49.592 | 66.865 |
| 4.0 mm | 55.284 | 4.151 | 54.396 | 4.879 | 49.737 | 65.450 |
| 5.0 mm | 54.883 | 3.665 | 54.183 | 4.377 | 49.897 | 63.664 |
| 6.0 mm | 54.622 | 3.438 | 53.993 | 4.039 | 49.774 | 62.888 |
| stromal | | | | | | |
| 2.0 mm | -3.470 | 0.401 | -3.368 | 0.537 | -4.433 | -2.917 |
| 3.0 mm | -3.440 | 0.341 | -3.367 | 0.440 | -4.238 | -2.967 |
| 4.0 mm | -3.418 | 0.304 | -3.361 | 0.395 | -4.124 | -2.989 |
| 5.0 mm | -3.377 | 0.252 | -3.333 | 0.328 | -3.969 | -3.009 |
| 6.0 mm | -3.346 | 0.227 | -3.306 | 0.289 | -3.877 | -3.021 |
| endothelial (posterior) | | | | | | |
| 2.0 mm | -7.058 | 1.119 | -6.718 | 1.617 | -9.594 | -5.556 |
| 3.0 mm | -6.987 | 0.949 | -6.708 | 1.364 | -9.087 | -5.747 |
| 4.0 mm | -6.904 | 0.818 | -6.697 | 1.149 | -8.711 | -5.808 |
| 5.0 mm | -6.747 | 0.639 | -6.603 | 0.849 | -8.207 | -5.869 |
| 6.0 mm | -6.643 | 0.549 | -6.536 | 0.700 | -7.951 | -5.870 |
| Supplementary Table 1: Front, stromal and back surface power in all eyes (D). The anterior surface power decreased from center to periphery (from 55.76 D at 2.0 mm to 54.62 D at 6.0 mm), consistent with a prolate anterior power profile.  The stromal and posterior powers, though negative, showed a similar flattening behavior with minimal changes across zones. Their contribution followed the same trend as the curvature, suggesting that these layers also maintain a prolate configuration overall.  Key finding: Surface power mirrors curvature (anterior positive, stromal/posterior negative), declining toward the periphery, consistent with a globally prolate profile. | | | | | | |
