# Supplementary material for: Stromal Curvature, Power and Corneal‐Stromal Curvature Ratios From a Hybrid AS‐OCT in Eyes With Keratoconus
Source: Clin Exp Ophthalmol. 2025 Sep 30;54(1):9–20. doi: 10.1111/ceo.70001 (PMC12886616; doi:10.1111/ceo.70001)
Supplement: Supplementary file 3 — Table S2: Central corneal (C), stromal (S), and epithelial (E) thickness in all eyes (mm). Central corneal, central stromal, and central epithelial thicknesses were consistent across zones. [file CEO-54-9-s001.docx]

| Values in mm | Corneal layer(s) | Mean | SD | Median | IQR | 95%CI lower bound | 95%CI upper bound |
| --- | --- | --- | --- | --- | --- | --- | --- |
| 2.0 mm | C | 0.492 | 0.049 | 0.496 | 0.065 | 0.394 | 0.583 |
|  | S | 0.440 | 0.047 | 0.442 | 0.062 | 0.343 | 0.529 |
|  | E | 0.052 | 0.007 | 0.052 | 0.008 | 0.039 | 0.068 |
| 3.0 mm | C | 0.493 | 0.048 | 0.497 | 0.065 | 0.400 | 0.584 |
|  | S | 0.440 | 0.046 | 0.443 | 0.062 | 0.344 | 0.529 |
|  | E | 0.052 | 0.007 | 0.052 | 0.008 | 0.040 | 0.067 |
| 4.0 mm | C | 0.493 | 0.048 | 0.497 | 0.064 | 0.401 | 0.584 |
|  | S | 0.441 | 0.046 | 0.443 | 0.062 | 0.347 | 0.529 |
|  | E | 0.052 | 0.007 | 0.052 | 0.008 | 0.040 | 0.067 |
| 5.0 mm | C | 0.495 | 0.047 | 0.498 | 0.062 | 0.406 | 0.584 |
|  | S | 0.442 | 0.045 | 0.444 | 0.060 | 0.352 | 0.529 |
|  | E | 0.053 | 0.006 | 0.052 | 0.007 | 0.041 | 0.067 |
| 6.0 mm | C | 0.496 | 0.046 | 0.499 | 0.062 | 0.408 | 0.584 |
|  | S | 0.443 | 0.044 | 0.445 | 0.059 | 0.354 | 0.530 |
|  | E | 0.053 | 0.006 | 0.053 | 0.007 | 0.042 | 0.066 |
| Supplementary Table 2. Central corneal (C), stromal (S), and epithelial (E) thickness in all eyes (mm). Central corneal, central stromal, and central epithelial thicknesses were consistent across zones. | | | | | | | |
